# A protocol for the Teen Bugs study: An integrative, multi-omics approach to understanding the role of the gut microbiome and mesocorticolimbic system in adolescent mental health following early adverse caregiving

**DOI:** 10.1016/j.bbih.2026.101275

**Published:** 2026-06-05

**Authors:** Genesis D. Flores, Zoe F. Damon, Mary Ford, Naomi N. Gancz, Paul W. Savoca, Shiba M. Esfand, Kristen A. Chu, Francesca R. Querdasi, Clare F. McCann, Jonathan G. Westman, Jennifer S. Labus, David Clewett, Ashley C. Parr, Elaine Y. Hsiao, Jonathan Jacobs, Jennifer Silvers, Bridget L. Callaghan

**Affiliations:** a Department of Psychology, University of California, Los Angeles, Los Angeles, CA, 90095, USA; b Lanser Westman Psychology Group, Los Angeles, CA, 90025, USA; c G. Oppenheimer Center for Neurobiology of Stress and Resilience at University of California, Los Angeles, Los Angeles, CA, 90095, USA; d Vatche and Tamar Manoukian Division of Digestive Diseases at University of California, Los Angeles, Los Angeles, CA, 90095, USA; e Goodman-Luskin Microbiome Center at University of California, Los Angeles, Los Angeles, CA, 90095, USA; f David Geffen School of Medicine at University of California, Los Angeles, Los Angeles, CA, 90095, USA; g Department of Psychiatry, University of Pittsburgh, Pittsburgh, PA, 15213, USA; h Department of Integrative Biology and Physiology, University of California, Los Angeles, Los Angeles, CA, 90095, USA; i Division of Gastroenterology, Hepatology and Parenteral Nutrition, Veterans Affairs Greater Los Angeles Healthcare System, Los Angeles, CA, 90075, USA

**Keywords:** Adolescence, Early adversity, Microbiome-gut-brain axis, Mesocorticolimbic system, Depression, Anxiety

## Abstract

Caregiving-related early adversities (crEAs) are potent risk factors for the development of internalizing psychopathology (e.g., depression, anxiety). Alterations to the dopaminergic mesocorticolimbic system, which supports the construction of reward-related experiences, are commonly observed following crEA exposure and are thought to mediate this risk. Indeed, many internalizing disorders are characterized by disruptions in how reward-related information is represented and used to guide affective and motivational states. Critically, the effects of crEA on mesocorticolimbic functioning may be shaped by input from peripheral systems, such as the gut microbiome, though such bottom-up signaling has been markedly understudied in humans. The Teen Bugs study was thus developed to identify gut microbiome-dependent metabolic pathways linking crEA exposure to mesocorticolimbic functioning and internalizing symptoms in adolescents, a group that experiences a disproportionate incidence of psychopathology relative to other age groups and is underrepresented in the gut microbiome literature. Adolescents aged 12–15 years, with and without histories of crEA exposure, will be followed across three timepoints over five years. At each timepoint, participants will complete a semi-structured clinical interview, a reward-guided decision-making task, and self-report questionnaires assessing mental health, previous caregiving experiences, reward-related behaviors, as well as developmental and lifestyle factors. Participants will also undergo multimodal neuroimaging that leverages MRI-based proxy markers of dopaminergic neurobiology and provide stool and blood samples for metagenomic and metabolomic profiling, respectively. This integrative design has the potential to clarify developmentally salient mechanisms that may serve as novel therapeutic targets for youth most at risk of, or already experiencing, internalizing psychopathology.

## Highlights

• Caregiving-related early adversity (crEA) increases risk for internalizing disorders.
• Internalizing disorders are linked to alterations of the mesocorticolimbic system.
• crEA has been shown to influence the development of the mesocorticolimbic system.
• The gut microbiome is shaped by crEA and implicated in mesocorticolimbic functioning.
• *Teen Bugs* tests gut-brain signaling pathways linking crEA to internalizing disorders.

**Table:** List of abbreviations (in alphabetical order):

**AAA**	Aromatic amino acid
**ADHD**	Attention-deficit/hyperactivity disorder
**BSS**	Bristol Stool Scale
**CBCL**	Child Behavior Checklist
**CLES**	Coddington Life Events Scale
**crEA**	Caregiving-related early adversity
**C-SSRS**	Columbia-Suicide Severity Rating Scale
**CTQ-SF**	Childhood Trauma Questionnaire – Short Form
**DA**	Dopamine
**DAergic**	Dopaminergic
**DCFS**	Department of Children and Family Services
**DSM-5 CCSM**	DSM-5 Cross-Cutting Symptom Measure
**FA**	Flip angle
**FAA**	Fatty acid amide
**FOV**	Field of view
**fMRI**	Functional magnetic resonance imaging
**KSADS-COMP**	Kiddie Schedule for Affective Disorders and Schizophrenia for School-Age Children, Computerized
**mPFC**	Medial prefrontal cortex
**MRI**	Magnetic resonance imaging
**NAcc**	Nucleus accumbens
**PAM**	Pubertal Appraisal Measure
**PDS**	Pubertal Development Scale
**PET**	Positron emission tomography
**PRTS**	Positive Risk Taking Scale
**PSS-10**	Perceived Stress Scale
**PVSS-21**	Positive Valence Systems Scale
**QUIC**	Questionnaire of Unpredictability in Childhood
**RCADS-25**	Revised Child Anxiety and Depression Scale
**REAP-S**	Rapid Eating Assessment for Participants – Shortened Version
**ROIs**	Regions of interest
**SCFA**	Short-chain fatty acid
**SHAPS**	Snaith-Hamilton Pleasure Scale
**SN**	Substantia nigra
**T**	Timepoint
**TE**	Echo time
**TR**	Repetition time
**V**	Visit
**VS**	Ventral striatum
**vmPFC**	Ventromedial prefrontal cortex
**VTA**	Ventral tegmental area

## 1 Introduction

Among the most salient influences on mental health across the lifespan are those embedded within early caregiving contexts. When caregiving quality or consistency is disrupted, the neurobiological programming of foundational cognitive, affective, and behavioral processes may be similarly compromised, predisposing youth to develop psychopathology (Callaghan and Tottenham, 2016; McLaughlin et al., 2017; Tottenham, 2012a). Caregiving-related early adversities (crEAs)—including caregiver-perpetuated maltreatment (e.g., abuse, neglect), prolonged or permanent separation from caregivers, and fragmented caregiving arrangements (e.g., foster or institutional care)—represent a class of disruptions that are alarmingly prevalent and well known to confer such developmental risks (Desmond et al., 2020; Finkelhor, 2020; Kessler et al., 2010; Yi et al., 2020). Of particular concern is the risk for internalizing disorders, such as depression and anxiety, as crEA-exposed youth report both clinical and borderline levels of internalizing symptoms more often than the general population (Heflinger et al., 2000; Moussavi et al., 2021). One possible explanation for these unfavorable odds is that crEAs significantly alter dopaminergic signaling within the mesocorticolimbic neural circuits that help the brain construct affective and motivational experiences—processes commonly dysregulated in depression and anxiety (Hanson et al., 2021). While evidence supports this theory, little is known about how crEAs come to produce neural circuit-level alterations that are associated with internalizing risk. As such, a shift away from models focused solely on the central nervous system toward more integrative frameworks that consider the role of peripheral systems in shaping neurodevelopment may be of critical value (Nusslock and Miller, 2016).

The gastrointestinal (i.e., gut) microbiome is one peripheral system with marked developmental plasticity and the capacity to modulate brain function via bidirectional signaling along the microbiome-gut-brain axis (Cryan and Dinan, 2012). These features render the gut microbiome a promising target for mechanistic inquiry and novel therapeutics (Borre et al., 2014). Accordingly, we designed the Teen Bugs study to delineate specific gut microbiome-dependent pathways linking crEA exposure to mesocorticolimbic system dysfunction and the incidence of depressive and anxious symptomatology across adolescence—a sensitive period for the onset of these disorders (Kessler et al., 2005). Although mesocorticolimbic alterations have been most consistently implicated in depression relative to anxiety, we will examine both given that they are highly comorbid in adolescents (Avenevoli et al., 2015).

In the sections that follow, we detail the rationale for the Teen Bugs study by synthesizing evidence linking crEAs to internalizing disorders, mesocorticolimbic system functioning, and the adolescent gut microbiome, as well as evidence implicating the gut microbiome in the dopaminergic activity of the mesocorticolimbic system. We will then outline the methods and procedures of the Teen Bugs study as a protocol to facilitate replication, collaboration, and secondary data analyses for what will be a highly multimodal and well phenotyped adolescent cohort.

### 1.1 crEAs and neurodevelopmental risk for internalizing psychopathology during adolescence

Caregivers are the most proximal and potent sources of environmental input during early development, providing scaffolding necessary for the adaptive organization of children's neurocircuitry (Gee and Cohodes, 2021). When that scaffolding is weakened, unstable, or entirely absent—as is the case with many crEAs—the brain may develop in maladaptive ways, or in a manner that is contextually adaptive but psychologically or metabolically costly over time (Callaghan and Savoca, 2026; Callaghan and Tottenham, 2016; Ellis et al., 2022). Indeed, varying forms of crEA (e.g., maltreatment, institutionalization) and associated experiential features (e.g., environmental unpredictability) have been linked to altered functional connections between the amygdala, hippocampus, prefrontal cortex, and striatum (Davis et al., 2025; Fareri et al., 2017; Gee et al., 2013; Hanson et al., 2018; Herzberg et al., 2021; Marusak et al., 2017; Silvers et al., 2016; for a review see Vannucci et al., 2023). These regions are central to cognitive-affective processing, and differences in their function are frequently implicated in internalizing psychopathology. Importantly, most internalizing disorders emerge during adolescence (Kessler et al., 2005), and the neural circuits that help support affective and regulatory capacities (e.g., frontolimbic, frontostriatal) undergo protracted maturational periods that extend into adolescence, rendering them vulnerable to the shaping effects of crEAs (Birnie et al., 2020; Birnie and Baram, 2022; Hoops and Flores, 2017; Reynolds and Flores, 2021). At the same time, this may position adolescence as a window of opportunity for salutary influences to redirect youth along more favorable trajectories of neurodevelopment (Méndez Leal and Silvers, 2021).

### 1.2 crEAs, reward-related behavior, and dopaminergic mesocorticolimbic system dysfunction

Altered motivational processes represent a common sequela of crEA that cut across internalizing psychopathologies (Dillon et al., 2014; Oltean et al., 2023). Compared to their unexposed counterparts, youth exposed to crEA often show slower reward-based associative learning and differences in reward-guided decision-making (Guyer et al., 2006; Hanson et al., 2017; Sheridan et al., 2018; Wismer Fries and Pollak, 2017; Xu et al., 2023). These behavioral differences are sometimes accompanied by blunted responsiveness to positive outcomes and heightened sensitivity to negative feedback (e.g., reward loss, punishment). Such response patterns can shape expectations about reward contingencies, reducing motivation to pursue rewards and biasing youth towards risk or uncertainty avoidance (Gerin et al., 2017; Humphreys et al., 2015; Kasparek et al., 2023; Kennedy et al., 2021; for a review see Smith et al., 2025), which notably map onto hallmark features of depression and anxiety. In other contexts, adversity-exposed youth have shown altered responsivity to, and less precise use of, risk and reward cues that manifest as impulsivity and a greater propensity for risk-taking (Birn et al., 2017; Guassi Moreira et al., 2022; Guyer et al., 2006; Oshri et al., 2018; Weller and Fisher, 2013). These tendencies may reflect adaptive strategies in environments where rewards are inconsistent or scarce (Burk and Averbeck, 2023; Nussenbaum and Hartley, 2024) but may otherwise be hazardous if chosen risks undermine well-being (e.g., substance misuse, self-injury).

Neurobiologically, crEA-induced differences in reward processing have been linked to alterations within dopaminergic (DAergic) mesocorticolimbic circuitry (Birnie et al., 2020; Hanson et al., 2021). This circuitry comprises DAergic neurons originating in the ventral tegmental area (VTA), which project to target regions in the ventral striatum (VS), prefrontal cortex, amygdala, and hippocampus (Beier et al., 2015). The nucleus accumbens (NAcc), within the VS, is often implicated in predictive processes that shape how cognitive, affective, and contextual information contribute to value construction for potentially rewarding and motivating stimuli (Bagot et al., 2015; Britt et al., 2012; Goto and Grace, 2008; Sesack and Grace, 2010). Studies using a variety of reward-based MRI tasks and resting-state approaches converge on the finding that childhood adversity predicts blunted activation of the VS/NAcc (Boecker et al., 2014; Goff et al., 2013; Hanson et al., 2015, 2016; Holz et al., 2017; Mehta et al., 2010; Richter et al., 2019), as well as increases in functional connectivity between the VS and anterior medial prefrontal cortex that appear age-atypical in youth (mPFC; Fareri et al., 2015; Fareri et al., 2017; Hanson et al., 2018). Critically, blunted striatal responses to reward are commonly associated with anhedonic symptoms and major depressive disorder (Borsini et al., 2020; Ely et al., 2021; Pizzagalli et al., 2009), suggesting that early adversity may shift predictive valuation in ways that heighten risk for mood disturbances. Moreover, mesocorticolimbic alterations in depression may be more pronounced during adolescence than adulthood, coinciding with marked developmental change and plasticity in this circuitry (Forbes and Dahl, 2012).

The aforementioned studies implicate crEAs in DA-sensitive circuit alterations that may elevate internalizing risk. However, it remains unclear how DA itself operates within these circuits across development and as a function of crEA exposure, as *in vivo* measurement of brain DA relies on invasive methods that are constrained in youth (e.g., positron emission tomography; PET). Adult PET studies nevertheless demonstrate increased striatal DA synthesis capacity and release following crEA (Egerton et al., 2016; Oswald et al., 2014; Pruessner et al., 2004; Soliman et al., 2008; for a review see Schalbroeck et al., 2024). Advancing our understanding of such associations in developing populations hinges on the use of innovative neuroimaging techniques that circumvent the limitations of PET and other invasive approaches for quantifying DA. Encouragingly, two proxy markers of DA neurobiology—striatal tissue iron concentration and neuromelanin—can be indexed in youth via MRI and will be measured in the Teen Bugs study (Cassidy et al., 2019; Parr et al., 2022; Peterson et al., 2018; Wengler et al., 2025).

### 1.3 crEAs and the adolescent gut microbiome

Over the past decade or so, the gut microbiome—the community of microorganisms residing within the gastrointestinal tract and their collective genetic material—has gained scientific prominence for its potential involvement in the etiology and pathophysiology of various psychiatric disorders, including depression and anxiety (Foster and McVey Neufeld, 2013; Kouraki et al., 2023; Malan-Muller et al., 2018; Nikolova et al., 2021; see Table 1 for definitions of key constructs in gut microbiome research used throughout this protocol). Beyond their established roles in aiding digestion, defending against pathogens, and regulating other aspects of physical health, some gut microbiota produce neuroactive metabolites and other signaling molecules that modulate central nervous system function via the microbiome-gut-brain axis, with implications for behavior and mental health (Cryan and Dinan, 2012; Miri et al., 2023). Critically, and much like the brain itself, the gut microbiome is highly plastic during early development, with sensitive periods that coincide with and may even precede those of the brain (Callaghan, 2020; Cowan et al., 2020). Because of this plasticity, the gut microbiome may act as an agent of developmental programming by which perturbations of the early environment (e.g., stress, antibiotics, malnutrition, etc.) can induce lasting alterations in microbial composition and function (for reviews see Green et al., 2025; Leigh et al., 2023; Ratsika et al., 2021). These alterations may, in turn, influence lifespan trajectories of neurodevelopment and psychiatric vulnerability.

**Table 1:** Key constructs in gut microbiome research and their definitions (in alphabetical order).

Construct	Definition
Alpha diversity	A within-individual measure of taxonomic diversity, with common metrics indexing richness (i.e., how many different taxa are represented in the gut) and/or evenness (i.e., how evenly those taxa are distributed)
Beta diversity	A measure of dissimilarity in taxonomic composition between individuals or groups
Differentially abundant	Taxa whose relative abundance, or representation in the gut, differs significantly between individuals or groups
Gut microbiome	The gut microbiota and their collective genetic material, including their genomes and gene products
Gut microbiota	The community of microorganisms (primarily bacteria but also archaea, fungi, and viruses) inhabiting the gastrointestinal tract, or gut
Taxa	Taxonomic groupings of microorganisms (e.g., phylum, class, order, family, genus, species) used to classify microbiota composition

Although knowledge in this field is advancing rapidly, most human research examining the role of the gut microbiome in mental health has focused its efforts on two extremes of the developmental spectrum: adulthood and, more recently, infancy through early childhood. Adolescence, by contrast, remains markedly understudied, despite being a period of dramatic structural and functional flux across biopsychosocial systems known to interact with the gut microbiome (Flannery et al., 2019; McVey Neufeld et al., 2023). Contrary to the long-held view that the gut microbiome stabilizes around the third year of life, emerging evidence suggests that the compositional and functional profiles of the adolescent gut microbiome are, in many respects, distinct from those of earlier and later life stages (Agans et al., 2011; Hollister et al., 2015; Radjabzadeh et al., 2020). Such distinctions thus warrant further investigation, especially in relation to crEA and age-related variation in its effects on the gut microbiome, as this may affect when risk-related phenotypes onset or worsen.

A small number of proof-of-concept studies have explored how crEAs impact the adolescent gut microbiome, offering preliminary support for crEA-induced programming effects on the gut microbiome that are detectable in adolescence. Indeed, relative to youth with no crEA exposure, previously institutionalized youth exhibited lower alpha diversity (Callaghan et al., 2020), group differences in overall community composition indexed by beta diversity (Callaghan et al., 2020; Reid et al., 2021), and differential abundance of specific taxa, including members of the order Clostridiales (Callaghan et al., 2020; Reid et al., 2021). Studies in younger children and adults exposed to more broad forms of childhood adversity have reported similar taxonomic and community-level differences (Hantsoo et al., 2019; Kazemian et al., 2024; Querdasi et al., 2023). However, all of these studies have relied on single timepoint assessments, limiting their ability to characterize microbial dynamics that may relate to the unfolding trajectory of internalizing symptoms that commonly follow adversity exposure. Moreover, these studies have all relied on 16S rRNA gene amplicon sequencing, further limiting insight into the functional capacity of the gut microbiome, which is critical for determining how internalizing risk would be instantiated. As such, longitudinal, mechanistically informed designs are a necessary next step, especially considering that the gut microbiome may influence the development and functioning of the mesocorticolimbic system.

### 1.4 The microbiome-gut-brain axis and mesocorticolimbic system functioning

**Fig. 1:**
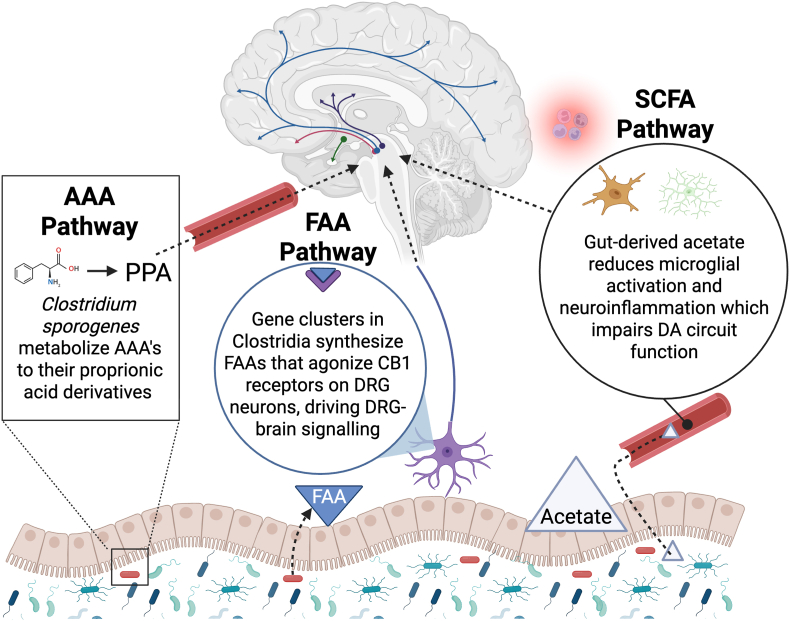
Mechanistic model of microbiome-gut-brain signaling pathways investigated in the Teen Bugs study. There are three metabolic pathways via which the gut microbiome can influence dopaminergic mesocorticolimbic system functioning: 1) aromatic amino acid (AAA) pathway, 2) fatty acid amide (FAA) pathway, and 3) short-chain fatty acid (SCFA) pathway. Abbreviations (in alphabetical order): CB1 = cannabinoid receptor 1; DA = dopamine; DRG = dorsal root ganglion; PPA = propionic acid.

In humans, studies are beginning to link specific taxonomic features of the gut microbiome to neural activity within the mesocorticolimbic circuit. For example, Dong et al. (2022) found that an elevated *Prevotella*-to-*Bacteroides* ratio was associated with high NAcc functional network influence (i.e., strong connectivity with other strongly connected, or influential, regions) in a sample of obese adults, who often show atypical processing of rewarding stimuli. Notably, this compositional profile was also associated with reduced levels of fecal tryptophan, an essential aromatic amino acid (AAA) processed by gut microbiota into various neuromodulatory metabolites. These findings are consistent with those of an earlier study that correlated gut microbiota-derived tryptophan metabolites with functional connectivity between the NAcc and other regions of the mesocorticolimbic system (Osadchiy et al., 2018). To date, only one study has examined these associations in youth. Specifically, in youth with attention-deficit/hyperactivity disorder (ADHD), a predicted microbial capacity for phenylalanine (another AAA and precursor to DA) synthesis was associated with NAcc activation to reward anticipation (Aarts et al., 2017). Together, these studies suggest that the gut microbiome may be capable of shaping mesocorticolimbic system development. This notion is further supported by a substantial body of rodent literature linking the gut microbiome with neurobehavioral responses to stimuli typically experienced as rewarding (de Wouters d’Oplinter et al., 2021; García-Cabrerizo et al., 2023; Jadhav et al., 2018; Kiraly et al., 2016; for a review see García-Cabrerizo et al., 2021). However, as in other areas of the gut microbiome literature, mechanistic understanding of that link in humans is limited by a reliance on solely taxonomic analyses achieved through 16S rRNA gene amplicon sequencing data. Meanwhile, rodent studies, leveraging metagenomic and metabolomic approaches, have mapped several pathways mediating gut-brain communication within DAergic mesocorticolimbic circuits with exceptional precision. These include pathways along which AAAs are metabolized into neuroactive derivatives, as well as those involving microbially synthesized fatty acid amides (FAAs) and short-chain fatty acids (SCFAs) that regulate neural and neuroimmune activity (Fig. 1). Critically, these pathways can be indexed non-invasively in humans, making them potential targets for developmental studies and the focus of the Teen Bugs study.

#### 1.4.1 Gut microbiome-derived targets linked to mesocorticolimbic system functioning

Dietary AAAs, namely tryptophan, phenylalanine, and tyrosine, are converted by gut microbiota into metabolites with demonstrated effects on neurotransmitter functioning, including monoamine (e.g., DA, norepinephrine, serotonin) functioning commonly implicated in depression, anxiety, and other internalizing disorders (Fernstrom and Fernstrom, 2007; Gao et al., 2020; Liu et al., 2020). Species within the genus *Clostridium* are primary contributors to this metabolic pathway, reducing AAAs to their corresponding propionic acid derivatives (Dodd et al., 2017; Elsden et al., 1976; Wikoff et al., 2009). These derivatives accumulate in host plasma (Wikoff et al., 2009) and, in some cases, enter the brain to stimulate DA release in the nigrostriatal DA pathway (i.e., DA projections from the substantia nigra [SN] to the dorsal striatum) that contributes to learning and motivational processes (Mangani et al., 2004). Therefore, alterations in the abundance or function of *Clostridium* species could likewise alter AAA metabolic output in ways that impair DAergic signaling, potentially impacting the development of valuation and motivational processes commonly dysregulated in internalizing disorders. This possibility is supported by a recent study showing that a phenylpropionic metabolite (3-[3′-hydroxyphenyl]propanoic acid) was enriched in mice transplanted with the fecal material of obese donors and further correlated with excessive motivation to obtain food rewards (de Wouters d’Oplinter et al., 2023).

Beyond AAA metabolism, rodent studies show that the gut microbiome can influence DAergic neural activity via the production of FAAs, which are lipid-derived signaling molecules with neuromodulatory potential (Ezzili et al., 2010). Interestingly, clusters of gene families within the class Clostridia (which includes *Clostridium*) seem to play a key role in the synthesis of FAAs (Chang et al., 2021). One study conducted in mice found that gut microbiome-dependent FAA metabolism stimulates peripheral cannabinoid receptors, which in turn drives dorsal root ganglion gut-brain signaling (Dohnalová et al., 2022). This neuronal activity was shown to limit exercise-induced monoamine oxidase-driven DA turnover in the striatum, resulting in greater synaptic DA and increased exercise motivation. A recent human PET study provides complementary evidence that FAA signaling is relevant for motivational functioning, though the study did not assess microbial FAA production. Specifically, greater availability of fatty acid amide hydrolase, an enzyme which degrades FAAs, in mesocorticolimbic regions was associated with greater apathy in adults with major depressive disorder (Rafiei et al., 2025). While these findings implicate FAA-mediated signaling in behaviors relevant to internalizing psychopathology, much remains to be understood about the upstream biological mechanisms through which this pathway is regulated in humans and across development, including the role of the gut microbiome.

Finally, SCFAs, including acetate, propionate, and butyrate, are a third class of neuromodulatory metabolites, produced through bacterial fiber fermentation, with members of the class Clostridia acting as dominant producers (Guo et al., 2020; Silva et al., 2020). Among their several functions, SCFAs influence neuroimmune processes, such as microglial activation (Caetano-Silva et al., 2023; Erny et al., 2015; Soliman et al., 2012). Critically, excessive microglial activation generates a pro-inflammatory milieu that has been associated with the expression of depressive- and anxiety-like behaviors across diverse rodent models (Wohleb and Delpech, 2017), which may be mediated, in part, by altered DAergic mesocorticolimbic system functioning. For example, rodent models of chronic pain and a prolonged high-calorie diet exhibited microglial activation and neuroinflammation within mesolimbic regions (i.e., VTA, NAcc), as well as disrupted DAergic signaling, which resulted in maladaptive behaviors toward reward-relevant stimuli (Gutiérrez-Martos et al., 2018; Taylor et al., 2015). Similar neuroimmune disturbances have been observed in the context of early life stressors, such as prenatal opioid exposure and adolescent social stress (Rodríguez-Arias et al., 2018; Smith et al., 2022). SCFA-dependent microglial modulation may therefore represent another pathway by which the gut microbiome shapes mesocorticolimbic system functioning across the lifespan.

## 2 The Teen Bugs study

The Teen Bugs study seeks to identify gut microbiome-dependent pathways that link crEA to developmental alterations in the DAergic mesocorticolimbic system and vulnerability to depression and anxiety throughout adolescence in a longitudinal cohort (12–15 years at enrollment, three visits each over five years). To address knowledge gaps left by prior research, the Teen Bugs study will leverage a multi-omics approach and innovative neuroimaging techniques that enable activity mapping across several targeted, mechanistic pathways which have been characterized with exacting detail in rodent models. Fecal metagenomics will allow for high-resolution profiling of gut microbiome composition at the species- or strain-level (exceeding that of 16S rRNA gene amplicon sequencing) and will provide insight into the functional potential of the gut microbiota. Plasma metabolomics will track circulating neuromodulatory metabolites which might have been produced by the gut microbiota and can provide a link to the brain. Novel MRI-based proxy markers of DA neurobiology will be measured alongside mesocorticolimbic circuit-level function to test pathways of gut-brain influence previously identified in rodent studies. Specifically, we will quantify striatal tissue iron, a cofactor for tyrosine hydroxylase (the rate-limiting step in DA synthesis) that co-localizes with DA vesicles and is concentrated in DA midbrain regions (Brass et al., 2006; Ortega et al., 2007). We will also assess neuromelanin, a darkly pigmented byproduct of catecholamine (e.g., DA, norepinephrine) metabolism that accumulates in the DAergic neurons of the SN and VTA (Clewett et al., 2016). Finally, assessments of risk taking and reward-guided decision-making, together with validated measures of depression, anxiety, and related disorders, will anchor microbial, metabolic, and neural data to observable behavior and clinical symptoms. With this integrative design, the Teen Bugs study represents the largest multimodal, hypothesis-driven examination of the gut microbiome as a predictor of mesocorticolimbic development and mental health outcomes in typically developing and at-risk youth to date. Our expected findings have the potential to inform novel, non-invasive treatments and preventative interventions for the many individuals who are experiencing, or are at risk of developing, affective and motivational disturbances following crEA exposure.

### 2.1 Study aims and hypotheses

In Aim 1, we will test whether crEA is associated, both cross-sectionally and longitudinally, with alterations in reward-related behaviors, functional connectivity within mesocorticolimbic circuitry, and indices of DA neurobiology. We hypothesize that relative to a comparison group, adolescents with a history of crEA will (H1) perform worse on a reward-guided decision-making task and (H2) show age-atypical frontostriatal circuit connectivity. We also hypothesize that the crEA group will exhibit (H3) lower striatal tissue iron concentration, a proxy marker of DA synthesis capacity, and (H4) lower neuromelanin signal in the VTA and SN, indexing DA metabolism. We further predict that crEA exposure will be associated with less developmental change in these outcomes across the three study timepoints (T1–T3).

In Aim 2, we will test whether crEA is associated, both cross-sectionally and longitudinally, with specific microbial taxa, functional pathways, and metabolic products implicated in DAergic mesocorticolimbic system modulation. Relative to the comparison group, we expect that crEA-exposed adolescents will have (H5) a lower abundance of *Clostridium* species in stool and (H6) corresponding reductions in circulating AAA-derived plasma metabolites, (H7) a lower abundance of FAA-producing gene clusters in stool and fewer FAA plasma metabolites, and (H8) a lower abundance of SCFA-producing gene clusters in stool and lower plasma concentrations of SCFAs. As in Aim 1, we expect crEA exposure to also be associated with less change in these outcomes from T1–T3.

In Aim 3, we will test whether reward-related behavior and mesocorticolimbic system function mediate the association between the gut microbiome and symptoms of depression and anxiety. We will also test whether these relationships are moderated by crEA exposure. We hypothesize that (H9) reduced activity within gut microbiome-dependent AAA, FAA, and SCFA signaling pathways will be associated with greater symptoms of depression and anxiety via their effects on reward-guided decision-making, frontostriatal circuit connectivity, and proxies of DA synthesis capacity and metabolism. Additionally, (H10) crEA exposure is expected to moderate these mediation pathways, with stronger indirect association observed among adolescents with histories of crEA. See Table 2 for a summary of outcomes for each aim and planned analyses.

**Table 2:** Study aims, primary outcomes, and corresponding measures and analyses.

H	Primary Outcome	Outcome Measure(s)	Analyses
**Aim 1:** Test whether crEA^a^ is cross-sectionally and longitudinally associated with alterations in reward-related behaviors (H1), mesocorticolimbic functional connectivity (H2), and indices of DA neurobiology (H3–H4)			
H1	Reward-guided decision-making	Map Reward Learning Task (during and post-fMRI scan)	**Planned:** We will apply LCA reGLMs to test group (crEA, no crEA) differences in the estimated means and trajectories of each outcome for H1–H4 *Potential covariates*: sex, age, education, BMI, pubertal stage (PDS, Tanner Line Drawings), menstrual cycle stage (Menstrual Health Questionnaire), hormonal contraceptive use **Exploratory:** We may expand analyses to other ROIs, consider voxel-wise connectivity, apply traditional activation GLMs, or use sPLS-DA/multilevel sPLS-DA to identify non-hypothesized discriminating neural phenotypes and profiles
H2	NAcc–vmPFC connectivity	Task-based fMRI	
H3	Striatal tissue iron concentration	Resting-state and task-based fMRI	
H4	VTA/SN neuromelanin signal	Neuromelanin-sensitive MRI	
**Aim 2:** Test whether crEA is cross-sectionally and longitudinally associated with specific microbial taxa (H5), metabolic products (H6–H8), and functional pathways (H7–H8) linked to DAergic mesocorticolimbic function			
H5	*Clostridium* species abundance	Shotgun metagenomics (stool sample)	**Planned:** We will apply LCA reGLMs to test group (crEA, no crEA) differences in the estimated means and trajectories of each outcome for H5–H8 *Potential covariates*: sex, age, BMI, diet (VioScreen, REAP-S), stool consistency (BSS), medication and supplement use **Exploratory:** We may test for non-hypothesized group differences in all identified metabolites using an untargeted platform, as well as in all gut microbiome species and gene pathway abundances using unsupervised learning (sPLS-DA and sPLS-regression), pathway enrichment analysis, and GLMs; we will also biobank fecal samples for follow-up fecal metabolomics
H6	AAA plasma metabolites	Metabolomics (blood sample)	
H7	FAA-producing microbial gene clusters; FAA plasma metabolites	Shotgun metagenomics (stool sample); metabolomics (blood sample)	
H8	SCFA-producing microbial gene clusters; SCFA plasma concentration	Shotgun metagenomics (stool sample); metabolomics (blood sample)	
**Aim 3:** Test whether reward-related behavioral and neural indices (Aim 1) mediate the association between gut microbiome-dependent metabolic pathways (Aim 2) and internalizing symptoms (H9), as well as whether crEA exposure moderates these associations (H10)			
H9	Direct and indirect effects of behavioral and neural indices on internalizing symptoms (depression/anxiety)	KSADS-COMP supplemented by RCADS-25, CBCL, and DSM-5 CCSM	**Planned:** We will apply longitudinal moderated mediation modeling via structural equation modeling to test H9; crEA will be specified as a moderator to test H10 *Potential covariates*: sex, age, education, BMI, pubertal stage, menstrual cycle stage, diet, stool consistency, hormonal contraceptive use, medication and supplement use **Exploratory:** We may derive crEA-associated signatures from gut microbiome and neural data using sPLS-DA to test in mediation models; we may also explore nonlinear changes over time
H10	Direct and indirect effects of behavioral and neural indices on internalizing symptoms (depression/anxiety) moderated by crEA	KSADS-COMP supplemented by RCADS-25, CBCL, and DSM-5 CCSM	

Note.

a crEA will be operationalized as reported history of foster or institutional care and supplemented by the Questionnaire of Unpredictability in Childhood and Coddington Life Events Scale. Abbreviations (in alphabetical order): AAA = aromatic amino acid; BMI = body mass index; CBCL = Child Behavior Checklist; crEA = caregiving-related early adversity; DA = dopamine; DSM-5 CCSM = DSM-5 Cross-Cutting Symptom Measure; FAA = fatty acid amide; fMRI = functional magnetic resonance imaging; GLMs = general linear models; H = hypothesis; KSADS-COMP = Kiddie Schedule for Affective Disorders and Schizophrenia for School-Age Children, Computerized; LCA = linear contrast analysis; MRI = magnetic resonance imaging; NAcc = nucleus accumbens; PDS = Pubertal Development Scale; RCADS-25 = Revised Child Anxiety and Depression Scale; REAP-S = Rapid Eating Assessment for Participants – Shortened Version; reGLMs = random effects general linear models; ROIs = regions of interest; SCFA = short-chain fatty acid; SN = substantia nigra; sPLS-DA = sparse partial least squares-discriminative analysis; vmPFC = ventromedial prefrontal cortex; VTA = ventral tegmental area.

### 2.2 Overview of study design and procedures

The Teen Bugs study will use a 5-year, accelerated longitudinal design to characterize gut microbiome-mesocorticolimbic system communication and mental health trajectories in a sample of adolescents with and without crEA exposure. Participants will have their data collected at three timepoints (T1–T3) spaced 18 months apart. Two visits (V1 and V2), spaced approximately 2 weeks apart, will occur at each of T1–T3. Prior to data collection, informed caregiver consent and child assent will be obtained by a research staff member. Caregiver consent will be obtained using a password-protected electronic consent form at T1, following a verbal review of study procedures. This consent will cover participation across T1–T3; however, study procedures will be re-reviewed with caregivers at each subsequent timepoint to confirm their continued understanding and willingness to participate. Child participants will receive an age-appropriate verbal explanation of study procedures, and verbal assent will be obtained at V1of each timepoint. Participants who reach the legal age of consent (18 years) during the study will be asked to provide informed consent via electronic consent form at their next scheduled visit.

**Fig. 2:**
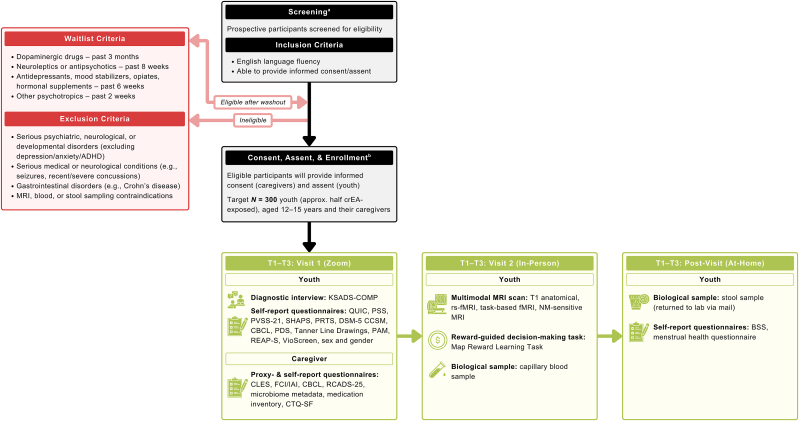
Flowchart depicting the Teen Bugs study protocol. Black boxes and arrows represent the flow of steps completed prior to and at enrollment. Red boxes and arrows represent how prospective participants will be screened out based on waitlist and exclusion criteria, with those waitlisted becoming eligible if medication washout periods have elapsed. Green boxes and arrows represent the flow of Visit 1, Visit 2, and post-visit activities completed at each study timepoint (T1–T3). ^a^Eligibility criteria will be re-confirmed at each subsequent timepoint. ^b^Caregiver consent will be obtained at T1 and apply to all subsequent timepoints, but youth assent will be obtained at each of T1–T3. Abbreviations (in alphabetical order): ADHD = attention-deficit/hyperactivity disorder; BSS = Bristol Stool Scale; CBCL = Child Behavior Checklist; CLES = Coddington Life Events Scale; crEA = caregiving-related early adversity; CTQ-SF = Childhood Trauma Questionnaire – Short Form; DSM-5 CCSM = DSM-5 Cross-Cutting Symptom Measure; FCI: Foster care inventory; IAI International adoption inventory; KSADS-COMP: Kiddie Schedule for Affective Disorders and Schizophrenia for School-Age Children, Computerized; MRI = Magnetic resonance imaging; PAM = Pubertal Appraisal Measure; PDS = Pubertal Development Scale; PRTS = Positive Risk Taking Scale; PSS = Perceived Stress Scale; PVSS-21 = Positive Valence Systems Scale; QUIC = Questionnaire of Unpredictability in Childhood; RCADS-25 = Revised Child Anxiety and Depression Scale; REAP-S = Rapid Eating Assessment for Participants – Shortened Version; SHAPS = Snaith-Hamilton Pleasure Scale. For interpretation of the references to color in this figure legend, the reader is referred to the Web version of this article.

At V1, a trained research staff member will administer a semi-structured clinical interview to participants via Zoom, evaluating symptoms of anxiety, depression, and related psychopathologies. Participants will then be asked to complete a battery of self-report questionnaires assessing their mental health, caregiving experiences, current life stress, reward-related behaviors and traits, pubertal development and experiences, dietary habits, demographic variables, and potential covariates (e.g., birth mode, breastfeeding history, anti-/pro-/prebiotic use). Caregivers of participants will also be asked to complete parallel proxy-report questionnaires at this visit, as well as one self-report questionnaire regarding their own experiences with childhood adversity. At V2, participants will undergo multimodal brain MRI scanning at the UCLA Ahmanson-Lovelace Brain Mapping Center. After scanning, participants will have their blood sampled for metabolomic profiling. They will additionally be provided with a stool sample collection kit and instructed how to use it for self-collection at home. Depending on participant availability and scheduling constraints, the order of V1 and V2 may be reversed, and the time between visits may exceed 2 weeks. See Fig. 2 for a timeline of study activities.

### 2.3 Recruitment of target sample

The target sample for the Teen Bugs study is 300 adolescents (aged 12–15 years at enrollment), half of whom have experienced some form of crEA (e.g., maltreatment, foster or institutional care; *n* = 150), and the other half being a comparison group of adolescents who have not been exposed to crEA (*n* = 150). Groups will be approximately matched on age and sex. Participants will be followed longitudinally across three study timepoints spaced approximately 18 months apart, resulting in a final age range of 12–18 years. Importantly, this age range covers maximal change in reward processing behavior, frontostriatal circuit connectivity, and striatal tissue iron concentrations.

Recruitment will occur through multiple channels to ensure a demographically diverse and representative sample of youth within both exposure groups. These include community outreach efforts (e.g., flyering in public spaces, participating in local community events, partnering with schools and organizations serving adoptive and foster families) and targeted advertising on social media platforms (e.g., Meta, Facebook groups). Participants will also be identified through research participant referral platforms (e.g., CliniContact), where families opt in to being contacted about the study, and through direct contact with well-patients in the UCLA Health system who meet study eligibility criteria.

#### 2.3.1 Sample size justification and power analysis

A power analysis was conducted using G∗Power (Faul et al., 2007, 2009) and was based on the primary outcomes for Aim 1, which examines the impact of crEA on mesocorticolimbic circuit connectivity (including NAcc connectivity with 5 ventromedial PFC [vmPFC] subregions during a reward-guided decision-making task), striatal tissue iron, and VTA/SN neuromelanin signal.

For between group comparisons (crEA-exposed vs unexposed) of the primary outcomes, a sample size of 150 participants per group (two-tailed, *α* = .05/7) provides 80% power to detect an effect size difference of Cohen's *d* ≥ .38, if it exists. Recent research relating crEA to mesocorticolimbic activation during viewing of happy versus scrambled faces in youth demonstrated an effect size difference of Cohen's *d* > .77 (Young et al., 2022). The proposed sample size also provides adequate power to detect associations as small as *f*^2^ = .03 (estimated using multivariate linear regression with 5 predictors, *N* = 300, *α* = .05) in mediation analyses. Further, it provides sufficient power to detect the anticipated effect size differences in gut microbiome (Cohen's *d* > .45–.98; Querdasi et al., 2023) and metabolomics data, as well as changes in edges of the interaction network. For integrative network analysis, we can detect correlations as small as *r* = .22 based on *n* = 150 per group.

### 2.4 Inclusion, exclusion, and waitlist criteria

Adolescents and their caregivers will be eligible to participate in the Teen Bugs study if they are able to understand and complete study procedures (i.e., fluent in English), and able to provide informed assent and consent. Exclusion criteria for adolescent participants include diagnosed psychiatric disorders other than depression or anxiety; diagnosed neurological or neurodevelopmental disorders other than ADHD; serious medical or neurological conditions (e.g., seizures, recent or severe concussions); gastrointestinal disorders (e.g., Crohn's disease, ulcerative colitis); and contraindications to MRI (e.g., pregnancy, braces, non-removable piercings), capillary blood sampling, or stool sampling.

Adolescents meeting any of the following criteria will be placed on a waitlist rather than excluded: having had an infectious illness within the past 2 weeks, or use of: DAergic drugs (e.g., metformin, apomorphine) within the past 3 months; neuroleptics or antipsychotics within the past 8 weeks; antidepressants, mood stabilizers, opiates, hormones (e.g. testosterone or steroid medications), or hormonal supplements (e.g., testosterone boosters) within the past 6 weeks; other prescription psychotropic drugs within the past 2 weeks (e.g., benzodiazepines, anxiolytics); or antibiotics or antifungals within the past 2 weeks. In the case of infectious illness, adolescents will be eligible to participate 2 weeks after the resolution of symptoms. Adolescents on the waitlist due to medication use will be eligible to participate once the medication's respective washout period has elapsed, provided that the medication was used temporarily and has since been discontinued, or that it is clinically safe and the adolescent and their caregiver are willing to pause use for their participation. Participants with ADHD who are prescribed DAergic drugs (e.g., amphetamine salts, methylphenidate), however, will only be asked to refrain from taking these drugs on the day of neuroimaging and biological sample collection. Participants with depression or anxiety will not be asked to discontinue any prescribed drugs, and their use will be recorded. Finally, because 2 weeks may not allow for complete restoration of the gut microbiome following antibiotic or antifungal use, we will record the type of antibiotic or antifungal used, as well as the date of completion, to control for time since last use in gut microbiome analyses.

### 2.5 Measures – questionnaires

#### 2.5.1 crEAs

*Questionnaire of Unpredictability in Childhood (Youth Self-Report)*. The Questionnaire of Unpredictability in Childhood (QUIC; Glynn et al., 2019) is a 38-item self-report measure of unpredictability in the early home and family environment. Items are divided into five subscales capturing variability in parental monitoring and involvement, parental predictability, parental environment, physical environment, and safety and security. Youth will be asked to indicate whether a presented statement was true (“yes”) or not true (“no”) for them prior to the age of 12 or 18, depending on the item. Higher total scores indicate greater overall exposure to childhood unpredictability, and higher subscale scores indicate greater unpredictability within the corresponding domain. In adolescents, the QUIC total score has shown strong internal consistency (*α* = .84); however, subscale scores have shown slightly lower alphas (*α*s = .42–.70; Glynn et al., 2019).

*Coddington Life Events Scale (Caregiver Proxy-Report)*. The Coddington Life Events Scale (CLES; Coddington, 1999) presents a list of 37 common and potentially stressful life events relevant to the child's developmental stage. These events largely concern changes in household and family dynamics, such as parental divorce, increased number of arguments with a family member, and the addition or loss of a family member. Caregivers will be asked to indicate whether any of the events have negatively impacted their child, and if so, at what ages. The CLES is among the most widely used measures of stressful life events in youth and has demonstrated predictive validity for later emotional and physical health outcomes (Compas, 1987).

*Foster Care and International Adoption Inventories (Caregiver Proxy-Report).* Caregivers who report that their child was adopted from foster care will be asked how many placements their child experienced and at what ages those placements occurred. Using this information, we will calculate the total number of caregiving transitions experienced across different ages. Caregivers who report that their child was adopted internationally will be asked to provide details about their child's early institutional care experience, including their child's age at placement and adoption, the quality of the institutional facility (e.g., cleanliness, building condition) and caregiving received, and the number of caregivers responsible for their child during placement.

*Childhood Trauma Questionnaire – Short Form (Caregiver Self-Report)*. The Childhood Trauma Questionnaire – Short Form (CTQ-SF; Bernstein et al., 2003) will be used to assess caregivers’ own experiences of childhood trauma and crEA. Caregiver history of such experiences has been associated with the intergenerational transmission of mental health risk in biological families (Racine et al., 2023), and emerging evidence suggests similar interactive effects in adoptive families (Leve et al., 2024), making it a relevant factor to examine within the present study. The CTQ-SF consists of 28 items divided across five subscales, including emotional abuse, physical abuse, sexual abuse, emotional neglect, and physical neglect. Caregivers will be asked to indicate the extent to which each item was true during their childhood on a 5-point scale ranging from 1 (“never true”) to 5 (“very often true”). Ratings will be summed to create a total score, with higher scores indicating greater trauma load. The CTQ-SF has demonstrated acceptable to excellent internal consistency across subscales in a community sample of adults (*α*s = .61–.92; Bernstein et al., 2003).

#### 2.5.2 Current life stress

*Perceived Stress Scale (Youth Self-Report)*. The Perceived Stress Scale (PSS-10; Cohen et al., 1983; Cohen and Williamson, 1988) is a 10-item self-report measure of recent subjective stress. Youth will be asked to rate the frequency of stress-related thoughts and feelings (e.g., perceived lack of control, inability to cope with personal problems) experienced over the past month on a 5-point scale ranging from 0 (“never”) to 4 (“very often”). Scores will be calculated by summing the ratings across all items, resulting in a total score ranging from 0 to 40. The PSS has demonstrated strong psychometric properties in the age range of adolescents included in this study (Marakshina et al., 2024; Whitney et al., 2022).

#### 2.5.3 Reward-related behaviors

*Positive Valence Systems Scale (Youth Self-Report)*. The 21-item version of the Positive Valence Systems Scale (PVSS-21; Khazanov et al., 2020) will be used to assess several reward-related subdomains of behaviors, such as desire for rewards, expectations of receiving rewards, willingness to expend effort to obtain rewards, anticipation of future rewards, and immediate and delayed response to rewards. Youth will be asked to rate the extent which each item was true of them over the past 2 weeks on a 9-point scale ranging from 1 (“extremely untrue of me”) to 9 (“extremely true of me”). Item ratings will be summed to create a total score and reward-related subscale scores, with higher scores indicating more positively-valenced reward responsiveness. While the PVSS-21 has not yet been validated in an adolescent sample, it has demonstrated excellent internal consistency in adults (*α* = .93) and acceptable to strong internal consistency across subscales (*α*s = .69–.88), as well as good test-retest reliability (Khazanov et al., 2020).

*Snaith-Hamilton Pleasure Scale (Youth Self-Report)*. The Snaith-Hamilton Pleasure Scale (SHAPS; Franken et al., 2007) is among the most widely used self-report scales of hedonic capacity. Across 14 items, youth will be asked to rate their ability to experience pleasure in everyday situations over the past few days (e.g., “I would be able to enjoy my favorite meal,” “I would find pleasure in small things, e.g., a bright sunny day, a telephone call from a friend”) on a 4-point scale ranging from 1 (“strongly disagree” to 4 (“strongly agree”). When scored dimensionally (Franken et al., 2007), total scores range from 14 to 56, with higher scores indicating greater symptoms of anhedonia. The SHAPS has demonstrated good internal consistency in an adolescent sample (*α* = .87; Leventhal et al., 2015).

*Positive Risk Taking Scale (Youth Self-Report)*. The Positive Risk Taking Scale (PRTS; Duell and Steinberg, 2020; Fischer and Smith, 2004) is a 27-item self-report measure that assesses positive (e.g., auditioning for a play, starting a friendship with someone new, enrolling in a challenging class) and negative risks (e.g., getting into a physical fight, cheating on a homework assignment or exam, skipping class). The PRTS has shown good internal consistency in 12–21 year olds (*α* = .82; Duell et al., 2022). For the present study, items related to sexual behaviors (e.g., “had unprotected sex,” “sent sexy messages or pictures to someone”) were removed due to California state laws deeming sexting among minors illegal and legally reportable.

#### 2.5.4 Adolescent mental health

*DSM-5 Cross-Cutting Symptom Measure (Youth Self-Report)*. Youth will report on recent psychiatric symptoms using the DSM-5 Cross-Cutting Symptom Measure (DSM-5 CCSM; Narrow et al., 2013). This 25-item measure assesses 12 transdiagnostic symptom domains, including depression, irritability, anger, mania, anxiety, somatic distress, psychosis, sleep disturbance, inattention, repetitive thoughts, substance use, and suicide/self-harm. Due to limitations in study staff support to address serious clinical concerns such as suicide and self-harm, items assessing these domains will be omitted in the present study, leaving 23 items. Youth will be asked to rate how often they have experienced or been bothered by each symptom over the past 2 weeks on a 5-point scale ranging from 0 (“not at all”) to 4 (“nearly every day”). Higher item-level ratings indicate greater symptom frequency and burden. Although the DSM-5 CCSM has not been formally validated in youth, many of its items are adapted from other psychometrically validated instruments (Clarke and Kuhl, 2014). Additionally, test-retest reliability for youth aged 11–17 years has been shown to range from good to excellent across most items (Narrow et al., 2013).

*Child Behavior Checklist (Youth Self- & Caregiver Proxy-Report)*. The 113-item Child Behavior Checklist for Ages 6–18 (CBCL; Achenbach and Rescorla, 2001) will be used to assess youth mental health and engagement in internalizing and externalizing problem behaviors. Both youth and their caregivers will complete the CBCL, rating the extent to which specific symptoms and problem behaviors have been characteristic of the youth over the past 6 months on a 3-point scale ranging from 0 (“not true”) to 2 (“very true or often true”). Item responses will be summed and sorted into six DSM-oriented scales—affective problems, anxiety problems, somatic problems, ADHD, oppositional defiant problems, and conduct problems—as well as into eight empirically-derived syndrome scales, which contribute to two higher order scales representing internalizing and externalizing problems. Higher scores indicate greater symptom severity and engagement in problem behaviors. The CBCL has demonstrated strong psychometric properties, with excellent internal consistency for the internalizing (*α* = .90) and externalizing (*α* = .94) scales, as well as good consistency across DSM-oriented scales (*α*s = .75–.84; Achenbach and Rescorla, 2001; Achenbach et al., 2003). As with the DSM-5 CCSM, items regarding suicidality and self-harm will be omitted from the CBCL. Modifications will also be made to items regarding gender non-conformance to ensure they assess clinically meaningful aspects of gender identity (see Esfand et al., 2024, for further details).

*Revised Child Anxiety and Depression Scale (Caregiver Proxy-Report)*. The 25-item version of the Revised Child Anxiety and Depression Scale (RCADS-25; Ebesutani et al., 2017) will be used to specifically assess youths’ anxious and depressive symptomatology via caregiver report. Caregivers will be asked to rate how often their child experiences each symptom on a 4-point scale ranging from 0 (“never”) to 3 (“always”). Ratings will be summed to create a total anxiety and depression score, with higher scores reflecting greater symptom frequency. The RCADS-25 has shown strong internal consistency for caregiver reports in both clinical (anxiety: *α* = .96, depression: *α* = .80) and school-based samples (anxiety: *α* = .94, depression: *α* = .79; Ebesutani et al., 2017).

#### 2.5.5 Puberty

*Pubertal Appraisal Measure (Youth Self-Report)*. The Pubertal Appraisal Measure (PAM; designed in-house by C.F.M.) is a mixed-method tool designed to assess individuals’ perceptions of their pubertal experiences. Youth will be asked to report whether they feel they began and are progressing through puberty earlier, later, faster, or slower than same-sex, same-grade peers, and to indicate how positively or negatively they feel about these comparisons on a 5-point scale ranging from 1 (“extremely negatively”) to 5 (“extremely positively”). Youth will also be asked to rate the extent to which various aspects of their lives (e.g., body image, self-perception, peer and family relationships, emotional control, romantic/sexual interest, and treatment by others) have changed since puberty began on a 5-point scale ranging from 1 (“no change at all) to 5 (“completely changed”). For each endorsed change, youth will indicate whether it was for the better or worse. At T3 only, youth will complete additional items estimating the age at which they and others first noticed their pubertal changes, as well items assessing contextual influences on their pubertal experiences (e.g., information and resources received, breadth of school-based education on puberty-relevant topics, preparedness, sources of support, and cultural or societal influences). Finally, open-ended questions will allow youth to share additional reflections.

*Pubertal Development Scale (Youth Self-Report)*. The Pubertal Development Scale (PDS; Petersen et al., 1988) will be used to assess youth's perceptions of their pubertal development across adrenarche, gonadarche, and growth domains. Separate versions will be administered to male and female youth. In both versions, youth will indicate the status of their growth spurt in height, body hair growth, and skin changes (e.g., pimples). The male version will additionally ask about the status of facial hair growth and voice changes, while the female version will ask about the status of breast development and whether they have begun menstruating. All status items will be rated on a 4-point scale ranging from 1 (“not yet started”) to 4 (“seems completed”).

*Tanner Line Drawings (Youth Self-Report)*. The Tanner Line Drawings (based on the Picture-based Interview about Puberty) depict different physical stages of pubertal development. Youth will be asked to select the picture most representative of their current developmental status. The scores derived can be separated into adrenal and gonadal processes, as well as an overall Tanner Stage. Following Shirtcliff et al. (2009), Tanner-like staging will be estimated from PDS items and combined with responses on the Tanner Line Drawings to create a composite index of pubertal development. Prior studies have used this method to improve the accuracy of self-reported pubertal staging (Barendse et al., 2022; Ellis et al., 2011; Ladouceur et al., 2019; Ravindranath et al., 2022), and earlier findings suggest strong agreement between the two measures within a single Tanner stage (Bond et al., 2006).

*Menstrual Health Questionnaire (Youth Self-Report)*. Coinciding with the day of their stool sample collection, youth who menstruate will be asked to report the first day of their last menstrual period, the typical length and month-to-month regularity of their menstrual cycle, which cycle day they are on, age at menarche, whether they are using any hormonal contraceptives (including oral contraceptives and contraceptive devices), and whether they are receiving any other hormone-based treatments.

#### 2.5.6 Diet

*Rapid Eating Assessment for Participants (Youth Self-Report)*. The Rapid Eating Assessment for Participants – Shortened Version (REAP-S; Segal-Isaacson et al., 2004) is a brief self-report measure designed to assess diet quality. The REAP-S consists of 16 items assessing weekly average intake of whole-grains, calcium-rich foods, fruits and vegetables, fat, cholesterol, sugar-containing foods, and sodium-rich foods. Youth will rate the frequency of their intake on a 3-point scale ranging from 1 (“never”) to 3 (“usually/often”). The version of the REAP-S used in this study was modified slightly by the research team for use with teens (e.g., changing the item “You usually feel well enough to shop or cook” to “A member of your family usually feels well enough to shop or cook”). Scores on the REAP-S have been shown to correlate with other validated diet quality measures and objective measures of diet quality from urine and plasma (Johnston et al., 2018).

*VioScreen (Youth Self-Report)*. The VioScreen is a web-administered food frequency questionnaire that assesses food consumption patterns and nutrient intake over the past 90 days. Aided by photographic representations, youth will be asked to select the foods and beverages they consume at least once a month and indicate their typical portion size. VioScreen has been validated for use with adults (Kristal et al., 2014) and has also been used for self-report with children as young as 6 years old (Deierlein et al., 2019). Both adult and youth participants in those studies rated VioScreen easy to complete via self-report.

#### 2.5.7 Potential covariates and metadata

*Sex and Gender Questionnaire (Youth Self-Report).* Youth will be asked to report their sex assigned at birth (i.e., female, male, intersex) and current gender identity (e.g., cisgender girl, cisgender boy, transgender girl, transgender boy, non-binary). Those who report a transgender identity will additionally be asked whether they have undergone or are currently undergoing a medical transition.

*Bristol Stool Scale (Youth Self-Report)*. The Bristol Stool Scale (BSS; O'Donnell et al., 1990) will be used to assess stool consistency. Youth, with assistance from caregivers as needed, will rate the consistency of their stool sample on a scale ranging from 1 (“firm”) to 7 (“watery”) using images and descriptions provided by the BSS. Youth will also be asked about how they were feeling on the day of collection, whether their diet was typical that day, and whether their sample consistency was typical for them.

*Microbiome Metadata (Caregiver Proxy-Report)*. Caregivers will be asked to report on environmental variables that may influence the gut microbiome, including: their child's birth method (cesarean or vaginal); their child's country of birth; whether their child was born prematurely; early life breastfeeding and weaning history; perinatal exposure to antibiotics; presence of pets in the home (past and current); exposure to farms and livestock; recent antibiotic, probiotic, or antifungal use; whether their child is currently following a special diet (e.g., vegetarian, gluten-free); and whether their child has ever received a gastrointestinal disorder diagnosis.

*Medication Inventory (Caregiver Proxy-Report)*. Caregivers will be asked to report on any medications or supplements their child is taking. For each medication or supplement listed, caregivers will be asked to provide the name, dosage, and duration of use by their child.

### 2.6 Measures – diagnostic interview

Current diagnoses of child psychopathology will be made for research purposes via a researcher-administered semi-structured clinical interview—the Kiddie Schedule for Affective Disorders and Schizophrenia for School-Age Children, Computerized (KSADS-COMP; Townsend et al., 2020). Specifically, research staff who completed standardized reliability training will administer modules assessing mood disorders, anxiety disorders, ADHD, and psychosis. Diagnoses, in accordance with DSM-5 criteria, will be reviewed by a clinically trained psychologist (B.L.C.).

### 2.7 Measures – reward-guided decision-making task

**Fig. 3:**
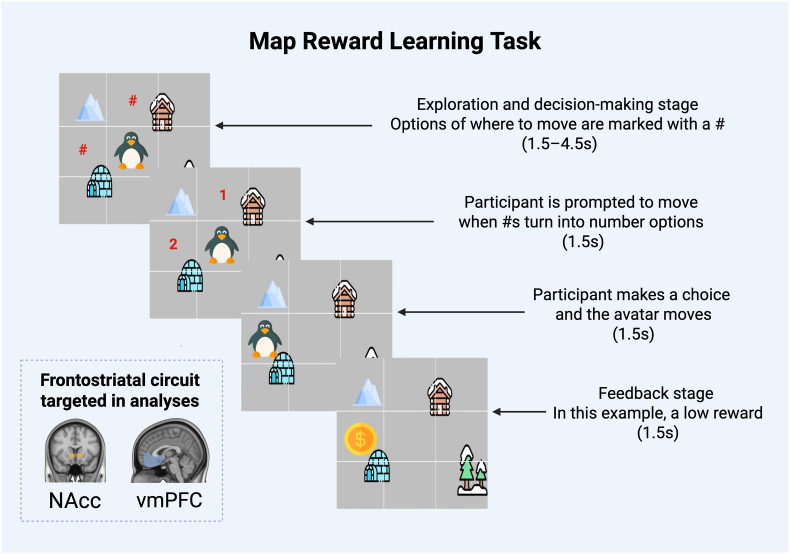
Example trial structure of the Map Reward Learning Task during the learning phase. Nucleus accumbens (NAcc) background functional connectivity with the ventromedial prefrontal cortex (vmPFC) will be the target of analyses.

Participants will complete the Map Reward Learning Task (Calabro et al., 2023; Esfand et al., 2024; Parr et al., 2021) in the MRI scanner and a test of their learning outside the MRI scanner. In this task, participants will move a cartoon penguin avatar around a landscape map to find monetary rewards, which are framed as performance-based incentives (up to $25). Participants will first be presented with a 3 x 3 grid ‘map’ that includes three cartoon landscape features (e.g., a mountain, an igloo). During the learning phase (inside the scanner)*,* participants will be instructed to explore the map to find the different monetary rewards (Fig. 3). In order to make their move, on each trial participants will be presented with a “#” in two of the grid squares, which indicate their movement options. After 1.5–4.5s “#” are replaced with a number “1” or “2”. Participants then select what position they would like to move to by pressing a button on the button box that corresponds to the number “1” or “2”. After selecting a number, the penguin moves to the corresponding area of the map, and a reward is then revealed if it is available. Each grid-square on the map had a fixed probability of giving a reward. If a reward is given, there is a 75% chance of a small reward (represented by a cartoon image of one coin) and a 25% chance of a larger reward (represented by a cartoon image of multiple stacked coins). Participants will complete two runs (36 trials each) during the learning phase.

Immediately after the MRI scan, participants will complete the test phase of the task. In this portion, participants will be asked to recall which areas of the grid map had the greatest number of rewards. To do this, participants will be presented with two options of grid squares to choose between, indicated by a “1” or “2” appearing in those squares on the map, and they will be asked to select the square which was associated with more and higher rewards. Once they make their selection, a “∗” will be presented in their selected square as visual feedback of their choice. All decisions are forced choice between two different reward probability squares (i.e., participants will never be asked to choose between two spots with equal chance of reward). The test phase contains a total of 27 trials and no feedback on accuracy of decisions is presented.

### 2.8 Measures – MRI sequences

All MRI scans will be acquired at the UCLA Ahmanson-Lovelace Brain Mapping Center on a 3T Siemens Prisma MRI scanner, using a 32-channel head coil.

#### 2.8.1 T1 anatomical sequence

High-resolution T1-weighted structural images, optimized for gray-white matter contrast, will be acquired using a magnetization-prepared rapid gradient-echo (MPRAGE) sequence with the following parameters: repetition time (TR) = 2300ms, echo time (TE) = 2.26ms, flip angle (FA) = 8°, in-plane resolution = 256 × 256, 192 sagittal slices; voxel size = 1.0 × 1.0 × 1.0mm.

#### 2.8.2 Functional MRI (fMRI) sequences

Functional images will be acquired in the transverse plane using an echo-planar imaging (EPI) sequence with the following parameters: TR = 1500ms, TE = 30.0ms; FA = 70°, field of view (FOV) = 192mm × 192mm, 36 slices; slice thickness = 3.0mm, voxel size = 2.0 × 2.0 × 3.0mm. Participants will first complete two runs of the Map Reward Learning Task, each acquired as a 4.5-min fMRI scan (173 vol). Then, a 7-min resting-state fMRI scan will be acquired (273 vol) as participants view *Inscapes*, an age-appropriate movie paradigm (Vanderwal et al., 2015).

Functional connectivity will be examined using both resting-state connectivity and task-based background connectivity approaches. Resting-state fMRI data will be analyzed in FSL to characterize functional connectivity in the absence of task demands (Jenkinson et al., 2012). Preprocessing will include motion correction, 4D slice-timing correction, skull stripping, intensity thresholding, wavelet despiking, coregistration to the participant's structural scan, nonlinear warping to MNI space, spatial smoothing, and intensity normalization. Bandpass filtering (.009 and .08 Hz) will occur simultaneously with a nuisance regression of head motion and non-gray matter signal. Volumes with frame-wise displacement >0.3mm will be excluded, and participants with at least 75% useable TRs will be retained.

Background connectivity, which is conceptually similar to resting-state connectivity but estimated during a task, will also be analyzed in FSL using data from the Map Reward Learning Task. Background connectivity analyses enable examination of persistent correlations across an entire psychological state (e.g., reward-relevant), rather than stimulus-evoked responses contrasted against a baseline, thereby reducing spurious correlations driven by shared stimulus-locked responses (Norman-Haignere et al., 2012). Preprocessing for background connectivity will follow the same pipeline as resting-state analyses. Then, both runs of task data will be concatenated, and trial level task-related BOLD responses will be regressed out of the time series using a TENT function. Model residuals will reflect BOLD fluctuations sustained throughout the task (i.e., background activity). Importantly, prior work using the same Map Reward Learning Task and other reward-relevant tasks has demonstrated that background connectivity measures are more sensitive to developmental changes across adolescence than resting-state connectivity measures, likely because they capture sustained mesocorticolimbic system engagement when the system is undergoing substantial maturation (Murty et al., 2018; Parr et al., 2021).

Background connectivity between the NAcc and vmPFC during the reward-relevant state, as well as resting state, will be modeled using FSL's FLAME 1 (Jenkinson et al., 2012). The NAcc will be seeded, and connectivity with the following five vmPFC regions of interest (ROIs) will be examined: posterior medial orbital frontal cortex, anterior vmPFC, ventral and rostral anterior cingulate cortex, subgenual cingulate (Mackey and Petrides, 2014). For each ROI pair, time series for reward-relevant and rest states will be created by taking the first principal component across all voxels within the ROI. This approach improves between region causal influence assumptions and decreases outlier voxel influence (Zhou et al., 2009). Bilateral NAcc-vmPFC Pearson correlation coefficients will be Fisher's r-to-Z transformed prior to group-level analysis.

Tissue iron will be estimated using the T2∗-weighted signal from the task and resting-state scans (Parr et al., 2022; Peterson et al., 2018). Importantly, this approach provides a non-invasive proxy of central DA that is a safe alternative to PET imaging in children. T2∗-weighted BOLD images will be minimally preprocessed, including 4D slice-timing and head motion correction, skull stripping, coregistration to the structural image, and non-linear warping to MNI space. Each volume will be normalized to the whole-brain mean, and the normalized signal will then be averaged across all volumes to generate a single T2∗-weighted image for each participant. High-motion timepoints will be identified as volumes containing frame-wise displacement > .3mm and will be excluded from analyses. Analyses will focus on mean T2∗-weighted signal extracted from bilateral NAcc ROIs, segmented using FSL FIRST, given the NAcc's relevance to mesocorticolimbic circuits and reward processing. Prior work shows excellent intraclass correlations in T2∗-weighted signal acquired across scans within a single session (Parr et al., 2022).

#### 2.8.3 Neuromelanin sequence

A T1-weighted fast spin echo sequence with the following parameters will be used to estimate neuromelanin: TR = 750ms, TE = 12.0ms, FA = 120°, FOV = 220mm × 220mm, 11 slices; slice thickness = 2.5mm; voxel size = .4 × 0.4 × 2.5mm; acquisition time = 3:44 minutes (Clewett et al., 2016). The FOV will be manually centered on the locus coeruleus (a critical supplier of norepinephrine), with a prescribed orientation encompassing the SN and VTA. As neuromelanin accumulates in the DAergic neurons of the SN and VTA, this neuromelanin-sensitive MRI sequence offers a novel, non-invasive approach for approximating DA function in developing populations (Cassidy et al., 2019; Wengler et al., 2025).

### 2.9 Measures – gut microbiome composition and function

#### 2.9.1 Stool sample collection

Stool samples will be collected by participants at home using OMNIgene®•GUT kits (DNAGenotek), with assistance from caregivers as needed. Participants will be instructed to deposit a small, pea-sized amount of stool into the collection tube, which contains a homogenization bead and stabilizing solution that preserve microbial DNA at ambient temperature for up to 60 days. This preservation method allows samples to be safely returned via mail without compromising their integrity. Upon receipt, samples will be aliquoted into sterile cryovials and stored in a −80°C freezer until they are ready for DNA extraction and sequencing.

#### 2.9.2 DNA extraction and sequencing

DNA extraction and sequencing will be performed at the UCLA Goodman Luskin Microbiome Center's Microbiome Core facility. Using PowerSoil Pro kits (Qiagen), DNA will be extracted from 100μl sample aliquots, following the manufacturer's instructions. Shotgun metagenomic libraries will be prepared with Illumina DNA Prep, and those passing quality control will be sequenced on the NovaSeq X Plus platform (2 × 150 bp paired-end reads, ∼20 million paired-end reads per sample, ∼6GB). Initial DNA extraction and sequencing will be performed on T1 stool samples. Once T2–T3 samples are collected, library preparation and sequencing will be performed on all T1–T3 samples, eliminating batch effects.

#### 2.9.3 Bioinformatics

Code used for preprocessing is available at https://github.com/bablab/bablab_hoffman_metagenomics and is derived from the bioBakery workflows (McIver et al., 2018). Briefly, KneadData (Liu et al., 2021) will be used for quality control, then HUMAnN (Beghini et al., 2021) and MetaPhlAn (Blanco-Míguez et al., 2023) will be used for functional and taxonomic profiling, respectively. For primary analyses, data will be filtered to remove very rare features and normalized using center-log ratio.

### 2.10 Measures – plasma metabolites

#### 2.10.1 Blood sample collection

After the MRI scan, a non-fasting capillary blood sample will be collected using the Tasso + device (Tasso, Inc.). The device will be applied to the participant's upper arm to collect 1000μl of blood into an EDTA-coated tube. A second device may be used if additional blood is needed to ensure sufficient sample volume, yielding a maximum total volume of 2000μl. Once collection is complete (or after 5 minutes has elapsed, whichever comes first), the device will be removed from the participant's arm, and the tube will be detached from the device and sealed.

#### 2.10.2 Plasma preparation

Whole blood will be centrifuged at 2000 RCF for 3 minutes to separate plasma. After centrifugation, the plasma supernatant will be aliquoted into sterile cryovials and stored in a −80°C freezer until samples are ready for mass spectrometry.

#### 2.10.3 Mass spectrometry

Mass spectrometry analysis will be performed through a commercial facility, using their proprietary platform. We will perform mass spectrometry on the T1 plasma first, preserving ∼50μl from each sample against which T2-T3 samples will be normalized, correcting for batch effects. In the unlikely case that insufficient plasma is available for normalization, we will use the company's well-characterized plasma pool.

Both untargeted and targeted plasma metabolomics will be performed. Untargeted metabolomics provides data outputs for more than 900 validated metabolites of diverse chemical categories, including FAAs and AAAs (the foci of this study), as well as bile acids, neurotransmitters (e.g. DA, GABA), endocannabinoids, and steroids, making them a rich source for later exploratory analyses. As SCFAs are unstable, a targeted SCFA panel (acetic acid, propionic acid, lactic acid, isobutyric acid, butyric acid, 2-methylbutyric acid, isovaleric acid, valeric acid, and caproic acid) will be used. Peaks will be quantified using area-under-the-curve. Missing values will be imputed using KNN imputation. Data will be log2-transformed and normalized for analysis. Multivariate outliers will be identified by evaluating the first and second principal components.

### 2.11 Risk assessment and response

Given that the population under investigation (i.e., adolescent youth, half of whom were exposed to crEA) is at high risk of experiencing psychopathology (Gunnar et al., 2007; Tottenham, 2012b; Vannucci et al., 2025), there is a chance that participants will spontaneously disclose or endorse information suggesting that they are at risk of harming themselves or others, or that they or another minor/dependent has experienced maltreatment. To ensure the safety of participants and others that may be implicated, research staff will follow a standardized risk assessment and response protocol. All research staff will be trained to identify verbal and behavioral indicators of risk, and electronic alerts will be programmed for select questionnaire items that may also signal risk.

If at any point during their participation an adolescent directly or indirectly discloses self-harm or suicidality, a research staff member will assess the immediacy and severity of risk by verbally administering the Columbia-Suicide Severity Rating Scale (C-SSRS; Posner et al., 2008) and consult with the principal investigator (PI) of the study, who is clinically trained. If the C-SSRS suggests moderate or high suicide risk, the staff member will notify the adolescent's caregiver, develop a safety plan with them, and provide a list of crisis and mental health resources. If suicide is imminent, and the adolescent is participating remotely (e.g., during a virtual visit – V1), the staff member will call 911 to request a welfare check; otherwise, the staff member will personally accompany the adolescent to the Ronald Reagan UCLA Medical Center emergency department. A similar protocol will be followed for disclosures of homicidal thoughts or intent. In these instances, the staff member will assess the immediacy and severity of homicidal risk and consult with the PI. If homicidal ideation, intent, or planning is active, the staff member will notify the caregiver and develop a safety plan with them. If homicide is imminent, the staff member will call 911.

Adolescents or their caregivers may also reveal maltreatment of the adolescent, their siblings, or other minors and dependents. Even if maltreatment occurred prior to adoption, was litigated, or historical in nature, research staff may still be required to act. If maltreatment is suspected, the staff member will ask clarifying questions that determine whether the child (or another child) is at current risk of harm by an adult. If they are, the staff member will gather as much relevant contextual information as possible, notify the PI and Child Protective Services, and submit a written report to the Los Angeles County Department of Children and Family Services (DCFS) within 24 hours. If not at current risk but past maltreatment is disclosed, the staff member will still gather information about the incident, consult with the PI and possibly Child Protective Services to determine whether a DCFS report is warranted, and submit one within 24 hours if so advised. Recognizing that instances of maltreatment and poverty are often conflated in mandated reporting practices, and that such practices have historically resulted in disproportionate harm to marginalized communities (Fluke et al., 2011), research staff will approach all disclosures with cultural humility and careful consideration of context.

## 3 Discussion

Approximately 50% of all internalizing disorders onset during adolescence (Kessler et al., 2005), with crEA exposure constituting a major risk factor for their development (Kessler et al., 2010). Despite the clear public health relevance of these disorders, up to 40% of adolescents do not respond to first-line psychotherapeutic and pharmacological interventions (Vitiello et al., 2011), highlighting a need for novel treatment approaches. Those targeting the gut microbiome offer significant promise for treating youth, as emerging evidence indicates that specific microbial taxa (e.g., Clostridia) and their metabolic derivatives (e.g., AAAs, FAAs, SCFAs) can modulate neural circuits implicated in internalizing psychopathology, including DAergic mesocorticolimbic circuits that support the construction of reward-related affective and motivational experiences (Aarts et al., 2017; Dohnalová et al., 2022; Dong et al., 2022; Osadchiy et al., 2018; Rodríguez-Arias et al., 2018; Smith et al., 2022). Microbial communities can also be non-invasively manipulated through a variety of methods (e.g., diet, prebiotics, probiotics), which have been shown to ameliorate depressive- and anxiety-like symptoms in many preclinical models of crEA exposure (Dandekar et al., 2022; De Santa et al., 2024; Peng et al., 2019; Zhu et al., 2022). However, before the success of such treatment approaches can be realized in humans, more detailed mechanistic information is needed on the specific pathways of microbiome-gut-brain communication that underlie youth internalizing psychopathology, for whom these pathways are most affected, and at what developmental stage. We designed the Teen Bugs study in hopes of generating this insight and, in doing so, bringing the field closer to targeted, developmentally informed interventions for youth most at risk of developing internalizing psychopathology.

Data collection for the Teen Bugs study will occur across three longitudinal waves over a five-year period, with interim analyses and dissemination. Anonymized non-genomic data (i.e., demographic, questionnaire, clinical, behavioral, neuroimaging, metabolomic, and gut microbiome taxonomic and community-level data) will be periodically deposited in the National Institute of Mental Health (NIMH) Data Archive (NDA). Genomic data (i.e., shotgun sequences of microbial genes) will be deposited in the Sequence Read Archive (SRA – National Center for Biotechnology Information). Additional data products, including cleaning, processing, and analysis scripts, and programmed tasks, will be uploaded to GitHub and Open Science Framework (OSF). By integrating open science practices into our study design, we aim to promote transparency and enable other investigators to interrogate the Teen Bugs dataset for research questions not addressed in this protocol.

### 3.1 Limitations and future directions

While the Teen Bugs study represents one of the most comprehensive mechanistic examinations of the microbiome-gut-brain axis’ role in youth mental health to date, and holds promise for informing novel treatment approaches for at-risk youth, it is not without limitations. First, the study uses an observational design, and although our longitudinal approach allows us to test prospective relationships between variables, causal inferences cannot be made. Experimental designs and intervention studies targeting the microbiome-gut-brain pathways outlined in this protocol will therefore be an important next step for establishing causal links to internalizing outcomes. Second, crEA will be operationalized primarily by endorsements of foster or institutional care history. Though we plan to supplement this with self- and caregiver report measures of other potentially adverse caregiving experiences using the QUIC and CLES, we may not fully capture the heterogeneity of crEAs experienced by this group. Additionally, crEA represents a potent form of early adversity with documented links to neural and microbial alterations in adolescent youth, but there are other adversities common among youth that may likewise affect our outcomes of interest in ways that our study is not designed to capture (e.g., community violence exposure, poverty). Future research should examine how these other forms and dimensions of early adversity affect the gut microbiome, its metabolic output, and mesocorticolimbic system functioning across adolescent development. Third, while we recognize that sex is an important biological variable with influences on both gut microbiome composition and mesocorticolimbic system development, our projected sample will not be sufficiently powered to detect biological sex differences in these outcomes. Our approach is therefore to include sex as a covariate in all primary analyses and to explore associations with sex, though future research should plan for adequately powered designs to formally test such sex differences. Finally, pubertal status will be assessed via youth self-report using the PDS and Tanner Line Drawings, rather than Tanner staging via physical examination by a trained professional. Self-report pubertal measures are common in developmental research and help minimize participant burden but may be less accurate than physical examination. Researchers interested in characterizing how puberty shapes microbiome-gut-brain signaling and risk for internalizing psychopathology should consider incorporating more precise, professionally-administered pubertal assessments.

## Funding

This research is supported by the National Institute of Mental Health of the National Institutes of Health (R01MH136259 to B.L.C. and F31MH139356 to N.N.G.) and by the National Science Foundation Graduate Research Fellowship Program (DGE-2034835 and DGE-2444110 to G.D.F. and P.W.S. and DGE-2146755 to S.M.E.). The content is solely the responsibility of the authors and does not necessarily represent the official views of the National Institutes of Health or the National Science Foundation.

## CRediT authorship contribution statement

**Genesis D. Flores:** Visualization, Writing – original draft, Writing – review & editing. **Zoe F. Damon:** Project administration, Writing – review & editing. **Mary Ford:** Project administration, Writing – review & editing. **Naomi N. Gancz:** Writing – original draft, Writing – review & editing. **Paul W. Savoca:** Visualization, Writing – original draft, Writing – review & editing. **Shiba M. Esfand:** Writing – original draft, Writing – review & editing. **Kristen A. Chu:** Project administration. **Francesca R. Querdasi:** Writing – original draft, Writing – review & editing. **Clare F. McCann:** Writing – original draft, Writing – review & editing. **Jonathan G. Westman:** Supervision, Writing – review & editing. **Jennifer S. Labus:** Resources, Writing – review & editing. **David Clewett:** Resources, Writing – review & editing. **Ashley C. Parr:** Resources, Writing – review & editing. **Elaine Y. Hsiao:** Writing – review & editing. **Jonathan Jacobs:** Resources, Writing – review & editing. **Jennifer Silvers:** Supervision, Writing – review & editing. **Bridget L. Callaghan:** Conceptualization, Funding acquisition, Methodology, Supervision, Visualization, Writing – original draft, Writing – review & editing.

## Declaration of competing interest

The authors declare that they have no conflicts of interest or relevant disclosures.

## Data Availability

No data was used for the research described in the article.
